# Supplementary material for: The Neonatal Environment and Health Outcomes (NEHO) Birth Cohort Study: Behavioral and Socioeconomic Characteristics and Drop-Out Rate from a Longitudinal Birth Cohort in Three Industrially Contaminated Sites in Southern Italy
Source: Int J Environ Res Public Health. 2021 Jan 30;18(3):1252. doi: 10.3390/ijerph18031252 (PMC7908468; doi:10.3390/ijerph18031252)
Supplement: Supplementary file 1 [file ijerph-18-01252-s001.pdf]

Inclusion criteria

The general criteria for combined residential and hospital-based recruitment of healthy pregnant women were:

- residence in a study area or a local reference area for at least one year;
- ability to speak and understand the Italian language;
- being 18-40 years old at the time of delivery;
- not following any program of assisted reproduction;
- absence of serious chronic diseases, such as diabetes, hypertension, etc.;
- absence of any evident complications during pregnancy diagnosed previous to signing informed consent.

Supplementary Table S1. Main characteristics of newborns and disease-specific outcomes investigated by means of questionnaire at different time points.

**Outcomes**	**Baseline**	**6 months**	**12 months**	**24 months**
Weight	X	X	X	X
Height	X	X	X	X
Head circumference	X			
Asthma		X	X	X
Allergy		X	X	X
Allergic diseases		X	X	X
Respiratory tract infections		X	X	X
Cognitive function				X
Language				X
ADHD				X
Autism				X
Sleep disturbances				X
**Maternal/child available covariates/ confounders**	**Baseline**	**6 months**	**12 months**	**24 months**
Home address	X	X	X	X
Breast feeding	X	X	X	X
Diet	X	X	X	X
Dietary supplements	X	X	X	X
Childcare attendance		X	X	X
Active smoking	X	X	X	X
Passive smoking	X	X	X	X
Alcohol consumption	X	X	X	X
Physical activity	X	X	X	X
Substance use	X	X	X	X
Medicine intake	X	X	X	X
Vaccinations				X
Air pollution	X	X	X	X
Access to green spaces		X	X	X
Water contamination	X	X	X	X
Pets	X	X	X	X
Use of electronic devices		X	X	
Cleaning products			X	X

Supplementary Table S2. Biological samples and analyses. Toxicological analyses included: polychlorinated biphenyl esters, dichloro-diphenyl trichloroethane, hexachlorobenzene, mercury, lead, cadmium, arsenic, zinc, copper, selenium.

**Biological samples**	**Time of collection**		**Downstream applications**
	**Baseline**	**24 months**	
Serum (maternal and cord)	X		Toxicological analyses, inflammatory markers (cytokines), miRNA
Plasma (maternal and cord)	X		Toxicological analyses, inflammatory markers (cytokines), miRNA
Urine (maternal and children)		X	Toxicological analyses
Hair (children)		X	Toxicological analyses
Nails (children)		X	Toxicological analyses
Placenta	X		Transcriptomics (mRNA, miRNA)
